# The effect of cathodal transcranial direct current stimulation during rapid eye-movement sleep on neutral and emotional memory

**DOI:** 10.1098/rsos.172353

**Published:** 2018-07-18

**Authors:** Jennifer M. Johnson, Simon J. Durrant

**Affiliations:** School of Psychology, University of Lincoln, Brayford Pool, Lincoln LN6 7TS, UK

**Keywords:** sleep-dependent consolidation, memory, tDCS, emotional, procedural

## Abstract

Sleep-dependent memory consolidation has been extensively studied. Neutral declarative memories and serial reaction time task (SRTT) performance can benefit from slow-wave activity, characterized by less than 1 Hz frequency cortical slow oscillations (SO). Emotional memories can benefit from theta activity, characterized by 4–8 Hz frequency cortical oscillations. Applying transcranial direct current stimulation (tDCS) during sleep entrains specific frequencies to alter sleep architecture. When applying cathodal tDCS (CtDCS), neural inhibition or excitation may depend on the waveform at the applied frequency. A double dissociation was predicted, with CtDCS at SO frequency improving neutral declarative memory and SRTT performance, and theta frequency CtDCS inhibiting negative emotional memory. Participants completed three CtDCS conditions (Theta: 5 Hz, SO: 0.75 Hz and control: sham) and completed an SRTT and word recognition task pre- and post-sleep, comprising emotional and neutral words to assess memory. In line with predictions, CtDCS improved neutral declarative memory when applied at SO frequency. When applied at theta frequency, no negative emotional word memory impairment was found but a positive association was found between post-stimulation theta power and emotional word recognition. SRTT performance was also not altered by either CtDCS frequency. Future studies should investigate overnight theta CtDCS and examine the effects of CtDCS during and after stimulation.

## 1. Introduction

Sleep facilitates memory consolidation [1–4], however, the mechanisms of this facilitation are debated[5–7]. The synaptic homeostasis hypothesis suggests that global synaptic downscaling occurs due to slow-wave activity (SWA) mainly during slow-wave sleep (SWS), restoring balance after synaptic potentiation during wake [8]. This downscaling can eliminate weaker memory traces [5]. The active systems consolidation hypothesis [7] suggests that SWA orchestrates the reactivation and reintegration of newly encoded information through interplay between the hippocampus and neocortex [6]. This dialogue involves slow-oscillatory (SO) neuronal activity which oscillates between hyperpolarizing ‘down-states’ and depolarizing ‘up-states’ [3]. During rapid eye-movement (REM) sleep, the created memory representation may be stabilized within pre-existing knowledge networks through synaptic consolidation [7]. Both hypotheses support a crucial role of SWA in memory consolidation.

High amplitude slow oscillations, typically less than 1 Hz frequency, dominate SWS [9] and play an important role in memory consolidation, reducing the amount of forgetting [1]. Performance benefits have been found for hippocampal-dependent tasks ranging from word list learning [10,11] to the serial reaction time task (SRTT) which contains declarative elements [12,13].

While SWS seems to benefit emotionally neutral memories, REM sleep is involved in consolidating emotional memory [14,15] with a greater benefit for negative than neutral items [16]. Theta band oscillations (4–7 Hz), which are characteristic of REM EEG, potentially modulate the relationship between REM sleep and preference for emotional memory consolidation [17].

Emotional memory is typically stronger than memory for neutral items [18,19], reflecting the co-activation of the hippocampus and amygdala when exposed to emotional stimuli [20]. The processing of emotional information is also influenced by the dorsolateral prefrontal cortex (DLPFC) [21–23]. A hemispheric asymmetry has been found in the DLPFC, with positive information being influenced by the left hemisphere and negative by the right hemisphere, known as the valence-specific hypothesis [24,25]. In line with this hypothesis, depressed individuals show a hypoactivity of the left with a concordant hyperactivity of the right hemisphere [26]. Attempts to restore the balance using electrical stimulation have yielded some positive results, suggesting a fruitful avenue of research [27].

It has also been established that transcranial direct current stimulation (tDCS) can influence memory [28]. tDCS is a non-invasive brain stimulation technique involving an anodal electrode to increase cortical excitability and a cathodal electrode to decrease/inhibit cortical excitability [29,30]. More recent research suggests that cortical changes could depend on the specific EEG waveform [31] and cortical folding in the brain, with either stimulation type resulting in mixed-field potentials [32]. Specifically, cathodal tDCS (CtDCS) electrically binds to SO down-states to hyperpolarize neurons while anodal binds to the up-states to depolarize neurons, but as SO down-states are already fully hyperpolarized tDCS can effectively only increase excitability even when cathodal [32]. However, as the theta waveform is not characterized by fully hyperpolarized down-states, instead acting as a ‘travelling wave’ [33], in principle, CtDCS can inhibit cortical excitability by further decreasing theta down-states, which may have a concomitant effect on emotional memory consolidation. tDCS may, therefore, influence memory differently depending on more factors than simply the form of the stimulation (cathodal or anodal).

A hemispheric lateralization effect has been observed when stimulating at theta frequencies, in keeping with the valence-specific hypothesis, such that the right hemisphere has preferentially improved negative memories [25]. However, bilateral stimulation has meant that the respective roles of anodal and CtDCS for memory alterations remain unclear [34]. The form of emotional stimuli used may also influence the effectiveness of applying tDCS to the DLPFC to alter emotional memory or valence, with some studies finding significant results for emotional images/faces [23]; Balzarotti & Colombo [34] and others find no significant results for emotion regulation [25]. No previous studies, however, have explored the influence of tDCS on emotional word stimuli, despite theta activity benefiting both memory for emotional images and emotional text [35,36]. Additionally, all studies to date have used tDCS during the encoding phase when examining emotional memory differences [25,37,38]. It may instead be beneficial to implement tDCS during memory consolidation, which could be more likely to influence later retention [7].

In recent times, tDCS has been applied during sleep to modulate memory consolidation, with differing levels of success [39,40]. It is currently debated whether or not emotional memory could be sufficiently altered during REM sleep, as no previous studies have examined this [28]. Previously, tDCS has been applied during REM sleep, with no apparent consolidation benefit for neutral declarative memories [41]. This may be a direct consequence of SWS preferentially consolidating neutral declarative memory [3]. In line with this, neutral declarative memory improved after the application of anodal SO (0.75 Hz) tDCS stimulation during SWS specifically to the prefrontal cortex (PFC) [42,43]. This improvement was attributed to enhanced spindle activity and an increased duration of SWS caused by tDCS entraining SO [44,45] potentially influencing the transfer and distribution of encoded information to the neocortex [28]. As SWS may also alter memory in the SRTT due to the presence of explicit learning [13], including a modulation of the PFC on task performance [46], entraining SOs through tDCS could, in principle, also influence consolidation of the SRTT. However, consolidation of the SRTT has also been shown to relate to REM sleep [47], so enhancement of theta power through tDCS could also potentially benefit the SRTT.

Applying tDCS during sleep entrains specific frequencies to alter sleep architecture [41,45], with a concomitant memory alteration [28] in some cases. This phenomenon has not been extensively studied when applying CtDCS at the SO and theta frequencies because it was seen as typically inhibitory [29] until the more recent theoretical developments which showed that excitation or inhibition can depend on the waveform of the applied frequency [32]. This study therefore aims to examine whether the effects of CtDCS on memory may depend on differences in waveform for each frequency. Applying CtDCS during REM at SO and theta frequencies is predicted to entrain the respective frequencies, subsequently enhancing and inhibiting memory, respectively, due to specific differences in the waveforms for each frequency.

Memory for neutral words is predicted to benefit due to the association between SOs and neutral declarative memory consolidation [6] and the fact that entraining SO with anodal tDCS applied at 0.75 Hz frequency improves hippocampal-dependent neutral declarative memory [41]. Memory for emotional words, and especially negative words, is predicted to be inhibited due to the cathodal stimulation at theta frequencies having the potential to inhibit neural activity [32,33] in the right hemisphere, which can influence specifically negative emotional memories [25]. However, these hypotheses have yet to be tested, because no study has previously examined tDCS for emotional word stimuli, no study has previously attempted to inhibit emotional memory using tDCS during sleep, and no study has directly compared the effects of stimulation at different frequencies during REM sleep on both neutral and emotional memory tasks. The SRTT could benefit from 0.75 Hz stimulation as performance improvement has been associated with the increased SWA seen in younger adults (compared with older adults) during SWS [48]. However, it has also been associated with changes in REM sleep [47], and seems to recruit declarative and procedural memory systems [46], so it may also benefit from 5 Hz stimulation.

This study will therefore test the hypothesis that CtDCS applied during REM sleep will enhance neutral hippocampal-dependent memory in both a word recognition task when applied at 0.75 Hz (boosting SOs), and inhibit emotional (and especially negative) memory in a word recognition task while having no impact on the SRTT when applied at 5 Hz (boosting theta oscillations). It will also examine whether or not stimulation at either frequency will affect performance in the SRTT. We chose to stimulate at theta frequency (5 Hz) during REM sleep, as suggested by Barham *et al*. [28], because theta oscillations during REM sleep have been implicated in emotional memory consolidation [17]. In order to evaluate the specificity of stimulation at the theta frequency, we also stimulated at SO frequency (0.75 Hz), as well as having a sham stimulation condition. The 0.75 Hz stimulation was also during REM sleep rather than non-rapid eye-movement sleep (NREM) sleep in which SOs usually occur in order to avoid confounding stimulation frequency and sleep stage and ensure that the comparison was specific to frequency differences. This is similar to the approach adopted by Marshall *et al*. [44], who stimulated at two different frequencies during NREM sleep.

## 2. Material and methods

### 2.1. Participants

Eighteen participants were recruited in total, of whom three had to be excluded due to equipment failure, leaving data for 15 participants (5 male, 10 female), aged 18–22 (20.73 ± 0.3; mean ± s.e.m.). Strict exclusion criteria included no history of sleep, neurological or psychological disorders and no current pregnancy, assessed by a self-report questionnaire. All participants were right-handed native English speakers. The study was approved by the School of Psychology Research Ethics Committee and the University of Lincoln, which is accredited by the British Psychological Society, and all participants gave informed consent and were free to withdraw at any time without penalty. Participants were offered course credit for their participation.

### 2.2. Stimuli

The stimuli were presented on an Acer Aspire F15 laptop with a 15.4^″^ screen, with responses given using numbers on the keyboard numeric keypad.

Matlab^TM^ R2016b (The Mathworks Inc., 2016) and the Psychophysics Toolbox-3 extension [49] were used for memory task presentation and to record/extract responses. Superlab^TM^ 5.04 (Cedrus Corporation, 2014) was used for SRTT stimulus presentation, with responses recorded into a text file. Matlab R2013b was used for triggering stimulation.

### 2.3. Emotional memory task

A word recognition task assessed memory for emotional and neutral words. Eight hundred and sixty four words were selected from the Norms of Valence, Arousal and Dominance for 13 915 English Lemmas database [50]. Words were placed into one of three emotion categories: positive, neutral and negative, based on their standardized assessed mean valence and arousal scores. The words were rated on emotional valence and arousal scales, respectively, with scores ranging from 1 to 9: 1 = highly negative, 5 = neutral, 9 = highly positive for valence and 1 = calming, 5 = neutral, 9 = exciting for arousal. Words in the positive, neutral and negative categories significantly differed in valence and arousal (*p* < 0.01 for all pairwise emotion combinations; table 1).

**Table 1.** Valence and arousal scores for each category. All values shown are mean ± s.e.m. *p*-values are based on pairwise comparisons.

	emotion			
	positive	neutral	negative	*p* (all pairs)
valence	7.65 ± 0.02	5.21 ± 0.18	2.12 ± 0.01	<0.01
arousal	4.85 ± 0.06	3.83 ± 0.04	5.25 ± 0.05	<0.01

The words selected were divided into three sets of 288, with 96 positive, 96 negative and 96 neutral words, one set for each session. From each set of words, 192 were used during the encoding phase (64 positive, 64 neutral and 64 negative) and 96 (32 positive, 32 neutral and 32 negative) were used in the retrieval phase as foils. The order of the word sets presented to participants during the encoding and retrieval phases were counterbalanced across tDCS conditions.

In the encoding session, participants were shown onscreen instructions informing them that the experiment is about to begin. A blank screen was then shown for 500 ms, followed by a central white fixation cross on a black background for 500 ms. The first word (text size 40 in Arial font) then appeared onscreen for 200 ms, followed by another blank screen for 500 ms and then a screen asking participants to rate word valence and word arousal on a nine-point scale ranging from very negative to very positive valence and very low to very high arousal using keyboard keys 1–9. This pattern then continued for each word, beginning with a central white fixation cross until all words were rated.

**Figure 1.**
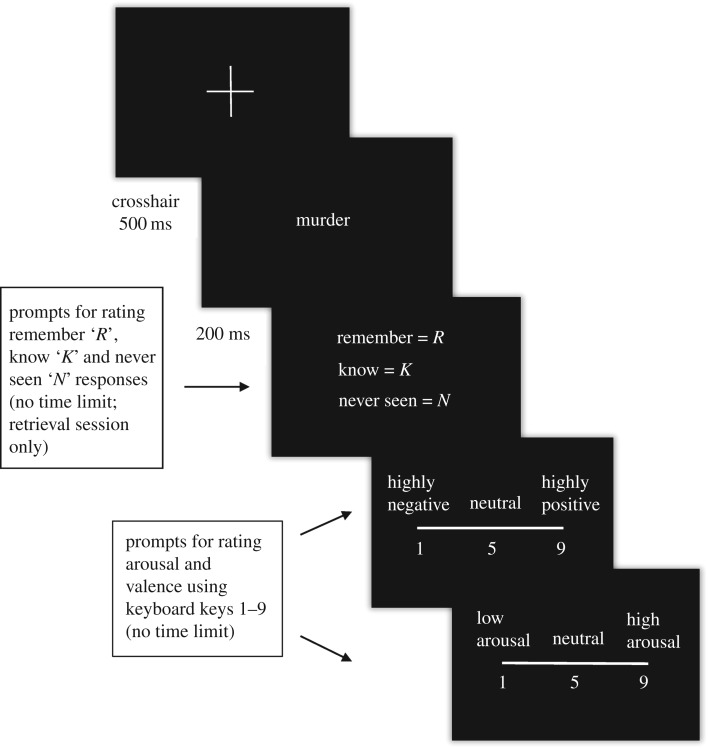
Example of a retrieval trial. Participants rated valence and arousal of the presented word on a nine-point scale as well as an ‘RKN’ memory judgement, with reaction times and accuracy being recorded.

In the retrieval session, a similar protocol to the encoding session was used with the addition of a memory rating for each word presented (figure 1). This involved making a memory judgement using the ‘RKN’ paradigm [51]. ‘R’ represented ‘Remember’ judgements, suggesting that the participant consciously remembered seeing the word in the encoding session, ‘K’ represented ‘Know’ that some memory of the contextual details of the word were known but not consciously remembered and ‘N’ represented ‘Never seen’ suggesting that the participant believed that the word was not presented in the encoding session.

### 2.4. Procedural learning task

**Figure 2.**
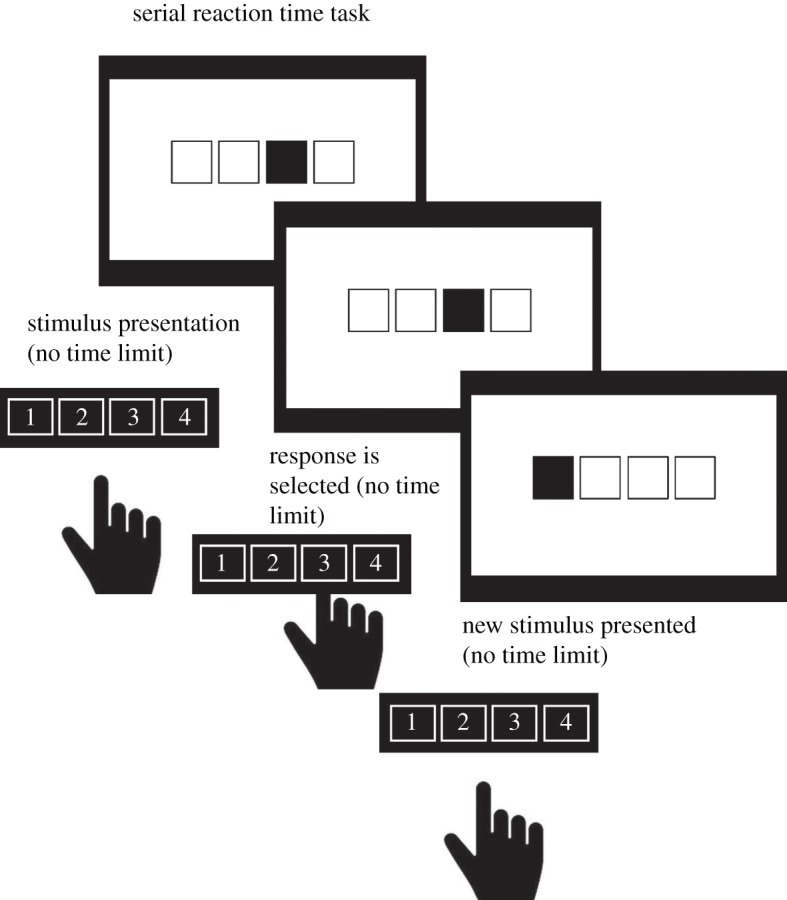
Example of a pre-sleep SRTT session. Participants pressed the key corresponding to the filled square onscreen, with accuracy and reaction times being recorded.

A four-choice serial reaction time task (SRTT) assessed procedural learning ability. Like the emotional memory task, the SRTT task involved sessions before and after sleep on each night of the study. Three SRTT sequences were created and counterbalanced between tDCS conditions and participants. The pre-sleep session consisted of an initial practice block of 12 trials (a trial being a single element within the sequence, hence a single repetition of the practice sequence), five control blocks with 96 trials each, one transfer block of 96 trials and finally two further control blocks of 96 trials each (figure 2). The post-sleep session consisted of an initial practice of 12 trials, followed by one control block of 96 trials, one transfer block of 96 trials and a final control block of 96 trials.

The control blocks contained a single, repeating, 12-element sequence; the transfer block contained a different 12-element sequence not previously learned by participants. The sequences consisted of the second-order conditional transitions (SOC); the target position on each trial depends on the target positions during the two previous trials [52]. The 12-element sequences were as follows based on previous sequences adopted in SRTT research; 342312143241 (SOC1), 341243142132 (SOC2) and 121342314324 (SOC3) [53].

Participants were instructed to press the key corresponding to the number shown on-screen during all blocks. If an incorrect response was made, a feedback beep was heard and the sequence would only continue upon pressing the correct key. On-screen instructions explained that the participant must respond quickly and accurately. Participants were not told about the presence of the sequences.

### 2.5. Equipment

#### 2.5.1. Transcranial direct current stimulation

A BrainSTIM^TM^ battery-driven direct current stimulator induced 0.4 mA cathodal pulse stimulation, the maximal current density achieved was 0.16 mA cm^−2^, which is in the middle of the 0.04 and 0.5 mA cm^−2^ range established by previous studies [41,54]. The tDCS stimulation frequencies used were 5 Hz (theta) and 0.75 Hz (slow oscillatory). During the sham session, the tDCS electrode was set up as usual but no stimulation was presented. During the stimulation period, a square-wave electrical pulse stimulation was turned on and off at the stimulation frequency, continually. A trigger box started the stimulation at the desired time using a custom-made Matlab script.

Stimulation was applied 4 min into REM sleep, lasting 25 min. As the second and third REM episodes are more stable [55], stimulation always occurred during the second or third REM periods [56]; the second REM period was used for all but one night, and the third used for just one night when the second REM period ended within the first 4 min. The use of a continuous stimulation period, similar to that used by Nitsche *et al*. [56], was chosen to ensure a stable block of stimulation which did not vary by structure or duration across participants. This is predicated on the assumption, however, that REM sleep continues through the stimulation period. This assumption is justified by the continued presence of REM sleep after the end of stimulation on 37 out of the 45 experimental nights; the eight nights in which REM ended during the stimulation were evenly split across the 5 Hz, 0.75 Hz and sham conditions (3-2-3) and exclusively in four participants. As all participants started stimulation during REM and ended at least one night during REM (and most all three nights), and as the pattern of results did not vary at all when the analysis was restricted to just the 11 participants who remained in REM throughout stimulation, data from all 15 participants is presented throughout.

Saline-soaked sponge electrodes covered in EC2^TM^ electrode cream to stabilize conductivity over time were used. Electrode resistance was always below 2 kΩ. The active stimulation cathodal electrode (2.5 cm²) was placed on F4 and the reference anodal (24 cm²) was placed on the contralateral upper forearm, making this CtDCS. The small cathodal electrode was placed on F4 in order to create focal stimulation in the right hemisphere. However, the anodal electrode was consequently too large (in order to maintain a safe current level [54]) to be placed near the EEG reference electrodes at the mastoids or the EOG electrodes in frontal orbital locations, without undue interference. Instead, as suggested by Reinhart *et al*. [57], we used an arm electrode in order to avoid unknown effects of stimulation from a large reference elsewhere on the scalp. This needed to be placed contralaterally in order to provide sufficient stimulation to grey matter which would not be achieved with safe levels of stimulation with the relative locations of our front lateral cathodal electrode and ipsilateral arm electrode. It should be noted, however, that this is likely to have decreased asymmetry in activation in comparison to an ipsilateral electrode.

#### 2.5.2. Polysomnography

Sleep monitoring involved an Embla^TM^ N7000 polysomnography system with scalp electrodes attached at international 10–20 system locations C3, C4, F3, O1 and O2 [58], in keeping with the recommended procedure from the American Academy of Sleep Medicine (AASM) [59]; the back-up electrode at F4 was omitted due to the presence of the stimulation electrode at that site. Each electrode was referenced to the contralateral mastoid (M1 and M2). Left and right electrooculogram (for eye movements) as well as left, right and upper electromyogram (for muscle tone) were also used, and a ground electrode was attached. Heart rate, respiration and movement were monitored through the attachment of the Patient Unit add-on device. Silver silver-chloride (Ag–AgCl) disc electrodes were attached to the scalp using EC2^TM^ electrode cream after skin exfoliation using NuPrep^TM^. Electrode impedance was below 5 kΩ for all electrodes in every recording.

#### 2.5.3. Procedure

**Figure 3.**
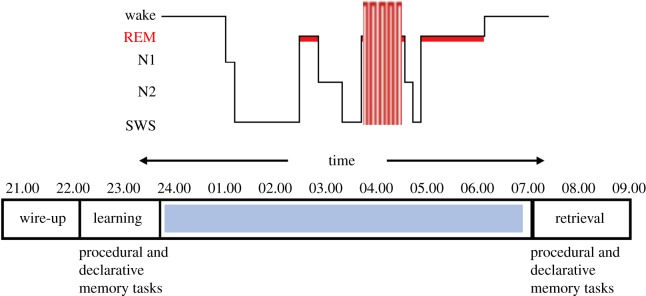
Depiction of experimental protocol. W, wake; REM, rapid eye-movement sleep; N1, N2: stages of non-rapid eye-movement sleep (NREM); SWS, slow-wave sleep.

The procedure is summarized in figure 3. All participants took part in the three CtDCS conditions (5 Hz, 0.75 Hz and sham), each session was separated by at least 48 h to resist potential CtDCS carry-over effects. Participants volunteered to take part in the study after briefing. The first session of the study began at 20.45, with participants giving informed consent and taking an opportunity to ask any questions. Participants then prepared for the overnight sleep before the wire-up and polysomnographic recording began.

After the wire-up, participants completed the Stanford Sleepiness Scale (SSS) [60] to record current alertness on a seven-point scale ranging from 1 (alert) to 7 (fighting sleep). At 22.00, this was followed by the pre-sleep session of the SRTT, lasting approximately 20 min and the encoding session of the word recognition task lasting approximately 40 min. At 23.30, lights were turned out and participants went to sleep. During the night, CtDCS stimulation was applied 4 min into either the second (mostly) or third (on one night only when the second period of REM was unstable in the first 4 min) stages of REM sleep in the night. Participants were awoken at 07.00 and given 20 min to recover from sleep inertia. Immediately after this, the SSS was administered again followed by the post-sleep session of the SRTT and word recognition tasks taking 20 min and 1 h, respectively. Finally participants were debriefed and left the laboratory at around 08.45.

### 2.6. Design and data analysis

#### 2.6.1. Recognition task

In order to focus on the strongest emotional memories, data analysis focused on the ‘Remember’ (R) responses. R-response rates were converted to d’ scores (d’ = Z(hit rate) − Z(FA rate)), this signal detection method accounts for possible response bias and is widely used in memory studies [61]. d’ scores were created for each emotion category. The relationship among emotion, CtDCS stimulation and memory was analysed using a 3 (emotion; positive, negative and neutral) x 3 (CtDCS condition; 5 Hz (theta), 0.75 Hz (slow-oscillatory) and sham (no stimulation)) within-subjects ANOVA and Bonferroni-corrected post hoc *t*-tests where appropriate.

#### 2.6.2. Serial reaction time task

SRTT measurements included accuracy (number of correct responses), response time (RT) and amount of learning (AoL), which is quantified as the mean RT in the transfer blocks − mean RT in the control blocks; hence a larger AoL represents a greater difference between learned and unlearned RTs. All RTs above and below 3 standard deviations from the mean were removed as outliers.

All analyses focused exclusively on blocks of interest, which were the transfer blocks (block 6 for pre-sleep sessions and block 2 for post-sleep sessions) and the control blocks immediately adjacent to the transfer blocks (blocks 5 and 7 for pre-sleep sessions and blocks 1 and 3 for post-sleep sessions). No other blocks were involved in the analysis. Transfer accuracy and RT measures were averaged across trials within the single transfer block while control accuracy and RT measures were averaged across trials from both control blocks of interest within a session. All measures were evaluated using a 3 (CtDCS condition; 5 Hz, 0.75 Hz and sham) x 2 (session; pre-sleep and post-sleep) within-subjects ANOVA with Bonferroni-corrected post hoc *t*-tests where appropriate.

#### 2.6.3. Sleep data analysis

Sleep data were divided into 30 s epochs and scored independently by two experienced sleep scorers using REMLogic^TM^ 1.1 (Embla, 2010). Using standardized scoring criteria from the AASM, a level of agreement of just under 85% was achieved, which is in line with other studies [59]. Total sleep time (TST), overall sleep efficiency (SE) and individual measures of duration and proportion were measured for each sleep stage (N1, N2, SWS and REM). The stimulation periods were marked as unscored because the stimulation frequency dominated the EEG during stimulation making reliable sleep scoring impossible; an equivalent period was also marked as unscored in the sham stimulation condition to allow valid comparison between the conditions. All sleep-related analyses have therefore been presented without the inclusion of the stimulation or sham-equivalent epochs. Planned Pearson's parametric correlation tests were conducted between the duration of each sleep stage and behavioural performance, for each CtDCS condition.

#### 2.6.4. Spectral analysis

The EEGLAB toolbox for Matlab [62] was used to manually remove artefacts in the EEG channels before spectral power was estimated using Welch's method with a 50% overlapping Hamming window using 30 s segments to match the scoring epoch length and a frequency resolution of 0.2 Hz [63]. Signals were high-pass filtered at just 0.1 Hz to remove the DC component without affecting SWA and total power from 0.5 to 90 Hz was estimated for all sleep epochs after the period of stimulation (or equivalent period for the sham condition). Activation was aggregated across all available electrodes and two bands of interest—SWA (0.5–2 Hz) and theta (4–8 Hz)—were defined in line with the stimulation frequencies using standard AASM definitions [59]. Relative power (as a percentage of total power) was calculated for each band separately for both REM and SWS; power estimation was limited to the post-stimulation sleep period in all cases in order to focus exclusively on the part of sleep that could have been influenced by CtDCS.

Four one-way within-subjects ANOVAs, for SWS SWA power, SWA theta power, REM SWA power and REM theta power, respectively, were used to analyse spectral power as a function of CtDCS condition. The relationship between spectral power and behavioural performance was evaluated using planned Pearson's correlations on specific variables of interest, separately for each CtDCS condition, based on theoretical predictions: SWA in SWS with neutral word recognition, theta power in REM with emotional word recognition, and both SWA in SWA and theta power in REM with AoL at retrieval. All other combinations were also tested as unplanned correlations with Bonferroni correction.

## 3. Results

### 3.1. Alertness

**Figure 4.**
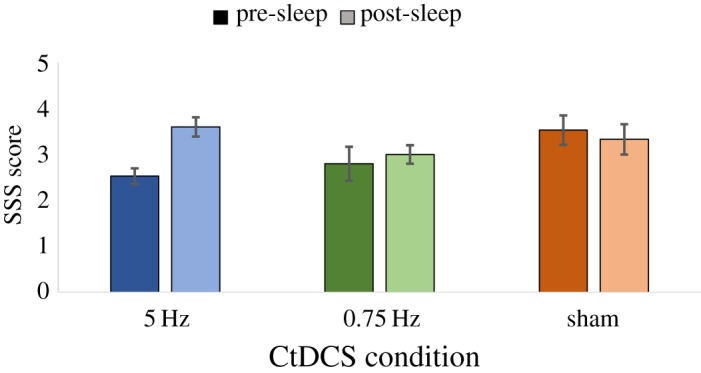
Alertness, measured by the SSS, shown before and after sleep in each CtDCS condition.

Alertness was measured pre-sleep and post-sleep in each CtDCS condition using the SSS to ensure that task performance was not impacted by differences in alertness; these results are shown in figure 4. Pre-sleep (2.96 ± 0.17) and post-sleep (3.31 ± 0.19) alertness did not differ overall (*F*_1,14_ = 3.47, *p* = 0.08, *η*_p_^2^ = 0.20) and alertness was also similar across the 0.75 Hz (2.90 ± 0.21), 5 Hz (3.07 ± 0.17) and sham (3.43 ± 0.21) conditions (*F*_2,28_ = 3.05, *p *= 0.06, *η*_p_^2^ = 0.18). In addition, pre- and post-sleep alertness differences did not significantly vary across CtDCS conditions (*F*_2,28_ = 2.36, *p *= 0.11, *η*_p_^2^ = 0.14). It is therefore unlikely that alertness played a major role in any of our findings here.

### 3.2. Word recognition task

**Figure 5.**
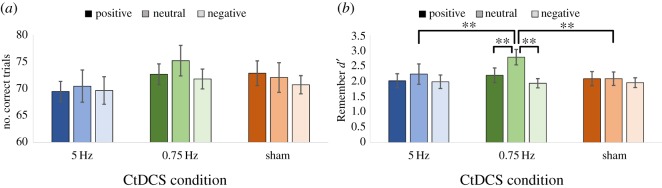
Performance on the word recognition task separately for each CtDCS condition and emotion category. (*a*) The number of correct trials; (*b*) Remember recognition performance measured by d’ (**p < 0.01).

The mean number of correct trials per session (figure 5*a*) was strongly above chance (214.98 ± 5.77), indicating a mean accuracy score of 74.6% when results were collapsed across CtDCS and emotion conditions. The number of correct trials did not differ significantly (*F*_2,56_ = 1.26, *p *= 0.30, *η*_p_^2^ = 0.08) between positive (71.67 ± 1.77), neutral (72.58 ± 2.49) and negative (70.73 ± 1.77) words, or differ significantly (*F*_2,56_ = 2.22, *p *= 0.13, *η*_p_^2^ = 0.14) between 0.75 Hz (73.22 ± 1.98), 5 Hz (69.87 ± 2.29) and sham (71.89 ± 2.12) conditions.

When comparing different CtDCS and emotion conditions for the ‘Remember’ response d’ (figure 5*b*), there was a non-significant main effect of CtDCS condition (*F*_1.32,18.51_ = 1.70, *p *= 0.21, *η*_p_^2^ = 0.11), although Bonferroni-corrected post hoc analyses suggested that the 0.75 Hz condition (2.30 ± 0.20) was significantly greater (*p *= 0.02) than the sham condition (2.04 ± 0.18), but not the 5 Hz (2.08 ± 0.24) condition. No other significant pairwise differences were seen (all *p *> 0.1). A significant main effect of emotion was found (*F*_2,28_ = 7.35, *p *= 0.03, *η*_p_^2^ = 0.34), indicating differences across the positive (2.10 ± 0.21), neutral (2.37 ± 0.22) and negative (1.95 ± 0.16) words. Post hoc analyses revealed a significantly greater d’ score for neutral than negative words (*p *= 0.004); no other pairwise comparison was significant (all *p *> 0.1).

It was predicted that 0.75 Hz CtDCS would improve memory most strongly for neutral words, while the 5 Hz CtDCS condition would decrease memory for negative words. A marginally significant interaction between CtDCS condition and emotion was indeed found (*F*_4,56_ = 2.47, *p *= 0.06, *η*_p_^2^ = 0.15). Planned comparisons showed that in the 0.75 Hz stimulation condition, memory for neutral words (2.78 ± 0.25) was significantly stronger than positive words (2.19 ± 0.24; *t*_14_ = 3.45, *p *= 0.004) and negative words (1.93 ± 0.15; *t*_14_ = 5.68, *p *< 0.001), in line with predictions. Notably, memory for neutral words was also significantly stronger for the 0.75 Hz condition (2.78 ± 0.25) than the sham condition (2.08 ± 0.22; *t*_14_ = 3.81, *p *= 0.002), confirming a specific effect of stimulation.

However, planned comparisons in the 5 Hz condition showed no significant difference between negative words (1.98 ± 0.22) and either positive words (2.01 ± 0.23; *t*_14_ = −0.218, *p *= 0.83) or neutral words (2.23 ± 0.33; *t*_14_ = −1.14, *p *= 0.27), contrary to predictions. There was no significant difference in negative word memory between the 5 Hz (1.98 ± 0.22) and sham conditions (1.95 ± 0.16; *t*_14_ = 0.204, *p *< 0.84). Collectively, these results show a clear benefit of stimulation at 0.75 Hz for neutral memory, but no effect of 5 Hz stimulation on negative memory.

Several quality control measures were taken to ensure the validity of the results. A comparison of word accuracy across the three different word lists used showed no significant differences (*F*_2,28_ = 0.28, *p *= 0.76, *η*_p_^2^ = 0.02) between word list 1 (216.33 ± 6.70), word list 2 (215.80 ± 7.85) or word list 3 (212.80 ± 4.43). Similarly, the word list order made no difference in the 0.75 Hz (*F*_5,9_ = 0.78, *p *= 0.59), 5 Hz (*F*_5,9_ = 1.93, *p *= 0.19) and sham (*F*_5,13_ = 0.98, *p *= 0.48) conditions. Finally, the order of the CtDCS conditions made no difference in the 0.75 Hz (*F*_5,9_ = 0.48, *p *= 0.79), 5 Hz (*F*_5,9_ = 0.28, *p *= 0.91) and sham (*F*_5,13_ = 1.65, *p *= 0.24) conditions.

### 3.3. Valence and arousal

**Figure 6.**
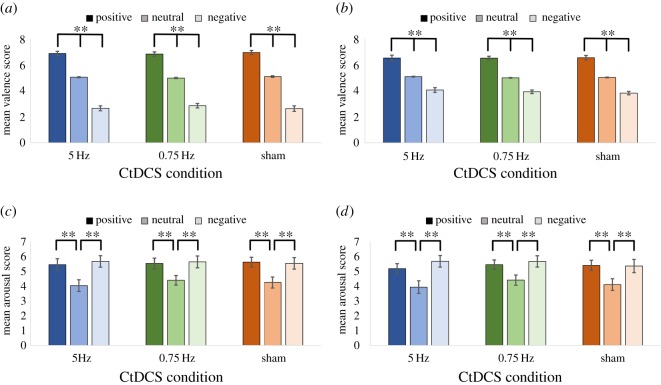
Valence and arousal ratings for encoding and retrieval separately for each CtDCS condition and emotion category. (*a*) Valence ratings during encoding; (*b*) valence ratings during retrieval; (*c*) arousal ratings during encoding and (*d*) arousal ratings during retrieval (**p < 0.01).

For valence ratings (figure 6*a,b*), positive valence was significantly stronger than neutral valence, which was significantly stronger than negative valence, in each session and across all conditions (all *p *< 0.001). For arousal ratings (figure 6*c,d*), positive and negative items created significantly stronger arousal than neutral items in each session and across all conditions (all *p *< 0.001); positive and negative items showed no difference in arousal to each in any session or condition (all *p *> 0.1)

The change in mean valence and arousal ratings from pre- to post-sleep was examined using difference scores [post-sleep valence/arousal − pre-sleep valence/arousal] as a function of CtDCS and emotion condition, in order to see if emotional reactivity was affected by stimulation (table 2). Positive difference scores indicate a more positive valence or a higher level of arousal after sleep.

**Table 2:** The difference in valence and arousal ratings across sessions, for CtDCS conditions within each emotion. All values shown are mean ± s.e.m*. p*-values represent the results of one-way ANOVAs comparing sleep parameters across tDCS conditions.

	condition		
	5 Hz	0.75 Hz	sham
positive words			
valence	−0.22 ± 0.11	−0.26 ± 0.09	−0.22 ± 0.13
arousal	−0.27 ± 0.16	−0.08 ± 0.08	−0.22 ± 0.08
negative words			
valence	0.27 ± 0.10	0.08 ± 0.11	0.11 ± 0.17
arousal	0.01 ± 0.08	0.03 ± 0.09	−0.18 ± 0.22
neutral words			
valence	0.04 ± 0.04	0.02 ± 0.03	−0.05 ± 0.05
arousal	−0.10 ± 0.08	0.10 ± 0.05	−0.14 ± 0.09

A significant main effect of emotion was found (*F*_1.16,16.2_ = 5.02, *p *= 0.04, *η*_p_^2^ = 0.26), with positive items (−0.23 ± 0.09) seen as marginally less positive relative to neutral items (0.01 ± 0.03; *p *= 0.07), and non-significantly less positive than negative items (0.15 ± 0.10; *p *= 0.2). There was no significant main effect of CtDCS condition (*F*_2,28_ = 2.55, *p *= 0.10, *η*_p_^2^ = 0.15) and no significant CtDCS × emotion interaction (*F*_2.36,33.0_ = 0.44, *p *= 0.68, *η*_p_^2^ = 0.03), suggesting that any change in emotional valence rating across the sleep interval was unrelated to the stimulation.

For arousal ratings, no main effect of emotion was found (*F*_2,28_ = 1.50, *p *= 0.24, *η*_p_^2^ = 0.10), with positive (−0.19 ± 0.06), negative (−0.05 ± 0.09) and neutral (−0.08 ± 0.04) items all showing a small decrease in arousal after sleep, with no differences between them (all *p *> 0.1). As with valence, there was no main effect of CtDCS condition (*F*_2,28_ = 1.00, *p *= 0.38, *η*_p_^2^ = 0.07) and no CtDCS × emotion interaction (*F*_4,56_ = 0.50, *p *= 0.74, *η*_p_^2^ = 0.03), so neither CtDCS condition nor word emotion affected any change in arousal ratings across sleep.

### 3.4. Serial reaction time task

**Figure 7.**
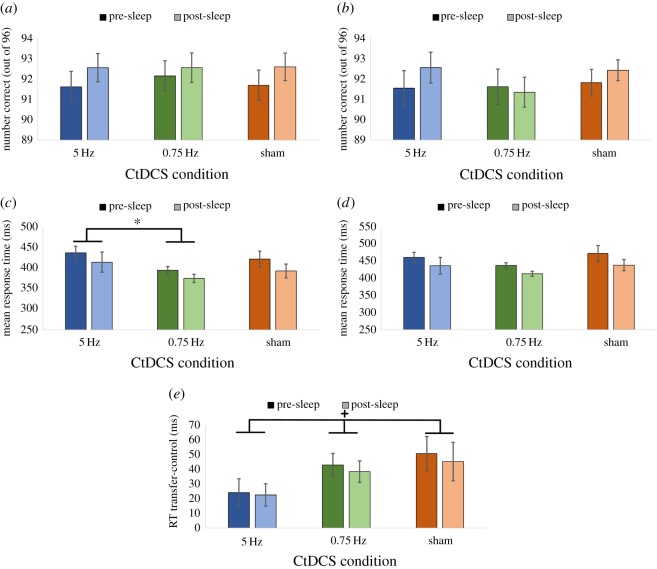
SRTT accuracy, response times and amount of learning separately for each CtDCS condition before and after sleep. (*a*) Accuracy in control trials; (*b*) accuracy in transfer trials; (*c*) response times in control trials; (*d*) response times in transfer trials and (*e*) amount of learning (*p < 0.05,+*p *< 0.01).

Accuracy (figure 7*a,b*) was assessed for the control blocks of interest as a function of each session (blocks 5 and 7 pre-sleep and blocks 1 and 3 post-sleep) and CtDCS condition, with no significant main effect of session (*F*_1,14_ = 2.64, *p *= 0.13, *η*_p_^2^ = 0.16) or CtDCS condition (*F*_2,28_ = 0.51, *p *= 0.61, *η*_p_^2^ = 0.04) or significant interaction (*F*_1.40,19.5_ = 0.23, *p *= 0.72, *η*_p_^2^ = 0.02). A similar analysis on the transfer blocks of interest (block 6 pre-sleep and block 2 post-sleep) also revealed no significant main effect of session (*F*_1,14_ = 0.60, *p *= 0.45, *η*_p_^2^ = 0.04), no significant main effect of CtDCS condition (*F*_2,28_ = 0.70, *p *= 0.51, *η*_p_^2^ = 0.05) and no significant interaction (*F*_2,28_ = 0.91, *p *= 0.41, *η*_p_^2^ = 0.06). Together these results suggest that any differences in reaction times (see next section) were unlikely to be due to differences in accuracy.

Reaction times (RTs, shown in milliseconds; figure 7*c,d*) were also examined as a function of session and CtDCS condition. For control blocks, a significant main effect of session (*F*_1,14_ = 4.99, *p *= 0.04, *η*_p_^2^ = 0.26) revealed a slight slowing from pre-sleep (394.67 ± 12.15) to post-sleep (418.53 ± 12.49). A marginally significant effect of CtDCS condition showed a marginally faster RT for the 0.75 Hz condition (385.27 ± 7.59) compared to the 5 Hz condition (426.38 ± 17.90; *p *= 0.098), with no significant difference from either compared to the sham condition (408.14 ± 17.63; both *p *> 0.1). The change in RT from pre-sleep to post-sleep did not vary by CtDCS condition (*F*_2,28_ = 0.13, *p *= 0.88, *η*_p_^2^ = 0.01). The slight slowing from pre-sleep (430.11 ± 11.27) to post-sleep (457.83 ± 12.40) was also seen in the transfer blocks (*F*_1,14_ = 6.65, *p *= 0.02, *η*_p_^2^ = 0.32). However, there was no difference across CtDCS conditions (*F*_1,14_ = 1.44, *p *= 0.26, *η*_p_^2^ = 0.09) and the change from pre-sleep to post-sleep again did not vary by CtDCS condition (*F*_1.41,19.78_ = 0.18, *p *= 0.76, *η*_p_^2^ = 0.01).

The amount of learning (AoL, the RT difference between transfer blocks and control blocks within a session; figure 7*e*) was also examined as a function of session and CtDCS condition. In all three CtDCS conditions within both sessions (six conditions in total) significant learning was demonstrated by faster RTs in control blocks in comparison to transfer blocks (all *p *< 0.05). AoL did not significantly vary (*F*_1,14_ = 0.46, *p *= 0.51, *η*_p_^2^ = 0.03) from pre-sleep (39.30 ± 6.02) to post-sleep (35.45 ± 6.56); however, it did vary marginally (*F*_2,28_ = 2.66, *p *= 0.09, *η*_p_^2^ = 0.16) across CtDCS conditions, with the greatest learning seen in the sham (48.04 ± 11.66) and 0.75 Hz (40.72 ± 6.41) conditions, and notably less in the 5 Hz condition (23.36 ± 6.20), though no pairwise differences were significant (all *p *> 0.1). The difference across CtDCS conditions did not vary significantly from pre- to post-sleep (*F*_2,28_ = 0.04, *p *= 0.96, *η*_p_^2^ = 0.003).

To ensure that none of the RT responses were due to the order in which the SRTT sequences were presented, we compared the effect of sequence order across all CtDCS conditions in both sessions, for both control and transfer blocks. No order effect was found (*p *> 0.1 for all 12 conditions), so SRTT sequence order is unlikely to be a major factor in our results.

### 3.5. Sleep parameters

Sleep parameter data separated by CtDCS condition are summarized in table 3. With standardized sleep scoring, and excluding the unscored stimulation period from all analyses, no differences were seen across CtDCS conditions for total sleep time, duration of N1 sleep, duration of N2 sleep, duration of SWS or duration of REM sleep (all *p *> 0.1), confirming that sleep structure was broadly equivalent across all three stimulation conditions. This suggests that the effect of stimulation did not significantly alter global sleep parameters outside of the stimulation period.

**Table 3:** Sleep parameters per CtDCS condition. All values shown are mean ± s.e.m. Higher scores equate to greater mean valence or arousal rating differences.

	CtDCS condition			
	5 Hz	0.75 Hz	sham	*p*
TST (min)	372.84 ± 15.28	375.93 ± 8.85	377.63 ± 9.21	0.88
SE (%)	90.04 ± 1.64	84.19 ± 2.55	90.91 ± 1.15	0.03
N1 (min)	10.17 ± 3.00	7.27 ± 1.30	7.67 ± 1.92	0.54
N2 (min)	165.33 ± 13.67	158.70 ± 17.01	184.50 ± 11.44	0.34
SWS (min)	115.87 ± 15.69	123.43 ± 14.38	111.47 ± 12.24	0.60
REM (min)	81.47 ± 8.34	86.53 ± 7.16	74.00 ± 7.00	0.15

In order to evaluate a role for different stages of sleep in the processing of emotional and procedural memory under specific stimulation conditions, we examined associations between the duration of different sleep stages and measures of behavioural performance. Planned correlation analyses between the duration of REM sleep in the 0.75 Hz, 5 Hz and sham conditions and the Remember d’ scores in the word recognition task in those conditions revealed no significant associations (all *p *> 0.1). Similar planned correlation analyses for SWS revealed a marginally significant relationship (*r *= 0.47, *p *= 0.08) between Remember d’ scores for positive words in the 0.75 Hz stimulation condition and the amount of SWS obtained in that condition. All other associations with SWS were not significant (all *p *> 0.1). Planned correlation analyses between the duration of REM and SWS in the 0.75 Hz, 5 Hz and sham conditions and AoL in the procedural learning task revealed no significant associations (all *p *> 0.1). Taken together, these results suggest that the benefit of 0.75 Hz stimulation for neutral words seen in the earlier analysis may be the result of the global increase in SWS for all participants as a result of the stimulation, and not related to the amount of SWS obtained outside of the stimulation period.

### 3.6. Spectral analysis

Relative spectral power was estimated for SWS and REM sleep during the post-stimulation period for both SWA and theta bands, as these are the sleep stages in which those bands are particularly important and the bands reflect the CtDCS stimulation frequencies used. Of the 45 experimental nights, SWS occurred at some point after stimulation on 42 nights (so three participants have no data on one night for this measure), and REM sleep occurred at some point after stimulation on all 45 nights as expected. The data are shown in table 4.

**Table 4:** Relative spectral power per CtDCS condition. All values shown are % ± s.e.m. Higher scores indicate a stronger proportion of spectral power in a given band of interest.

	CtDCS condition			
	5 Hz	0.75 Hz	sham	*p*
SWS SWA	75.62 ± 2.28	76.30 ± 2.38	74.21 ± 2.55	0.81
SWS theta	6.41 ± 0.76	6.54 ± 1.05	7.33 ± 0.96	0.61
REM SWA	51.25 ± 2.05	55.01 ± 2.71	51.48 ± 2.32	0.14
REM theta	14.52 ± 0.90	13.38 ± 1.14	15.06 ± 1.01	0.16

**Figure 8.**
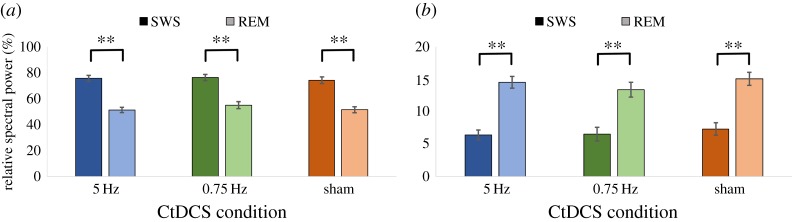
Relative spectral power during SWS and REM sleep separately for each CtDCS condition. (*a*) Relative power in the SWA (0.5–2 Hz) band; (*b*) relative power in the theta (4–8 Hz) band (**p < 0.01).

Relative power in the SWA band figure 8*a* during SWS was much higher than during REM across all conditions as expected (all *p *< 0.01). Similarly, relative power in the theta band (figure 8*b*) was greater during REM sleep compared with SWS across all conditions as expected (all *p *< 0.01). However, there was no difference in relative SWA power across CtDCS conditions during SWS (*F*_2,22_ = 0.21, *p *= 0.81, *η*_p_^2^ = 0.02), or during REM sleep (*F*_1.30,14.30_ = 2.38, *p *= 0.14, *η*_p_^2^ = 0.18). A similar analysis of relative theta power across CtDCS conditions revealed no difference during SWS (*F*_1.30,14.30_ = 2.38, *p *= 0.14, *η*_p_^2^ = 0.18) or during REM sleep (*F*_1.30,14.30_ = 2.38, *p *= 0.14, *η*_p_^2^ = 0.18). Collectively, these results suggest that stimulation at different frequencies did not have a major lasting impact on sleep EEG beyond the period of stimulation itself.

The association between theta power in post-stimulation REM and emotional memory was examined with planned correlation analyses. During in the 5 Hz (theta) stimulation condition, a positive correlation with theta power was found for both positive (*r *= 0.55, *p *= 0.04) and negative (*r *= 0.53, *p *= 0.04) emotional memory. No such correlation was seen in the 0.75 Hz stimulation condition (both *p *> 0.1) while in the sham condition, a trend was seen for negative emotional memory (*r *= 0.47, *p *= 0.08). These results suggest that the relationship between post-stimulation REM theta power and emotional memory may have been tightened as a result of the theta stimulation. By contrast, no relationship was seen between neutral memory and SWA power during SWS in any condition (all *p *> 0.1).

The AoL in the SRTT task could be governed by SWA during SWS or theta activity during REM sleep based on the literature, so planned analyses examined both of these possibilities. In the theta stimulation condition, the association between theta power during REM sleep and the AoL after sleep showed a trend (*r *= 0.46, *p *= 0.09). No relationship was found with SWA during SWS, or for either sleep stage in regard to the AoL before sleep.

Finally, all other possible relationships between behavioural measures and SWA power and theta power in either SWS or REM sleep, which were without theoretical predictions, were tested using Bonferroni-corrected Pearson correlation analyses. No significant effects were found (all *p *> 0.1).

## 4. Discussion

This study investigated the role of CtDCS applied during REM sleep at 0.75 Hz (SO) and 5 Hz (theta) frequencies in the consolidation of neutral and emotional memory in a word recognition task, and performance on the SRTT. CtDCS was applied during REM to entrain SO and theta oscillations—with SO as the hallmark of EEG activity during SWS being associated with neutral declarative memory especially [64] and theta oscillations as the hallmark of EEG activity during REM being associated with emotional memory [17]. Specifically, this study aimed to test a possible double dissociation by using CtDCS to impair negative memory consolidation when entraining theta oscillations while reducing forgetting of neutral declarative memory when entraining SO. It also aimed to examine the effect of stimulation at both frequencies on the SRTT which has been associated with both SWS and REM.

In line with predictions, after applying 0.75 Hz (SO) frequency CtDCS during REM sleep, a significant improvement in neutral declarative memory was seen relative to the sham and 5 Hz conditions. However, 5 Hz (theta) stimulation during REM sleep did not appear to significantly reduce consolidation of negative emotional memories. No effects of CtDCS were seen on the valence and arousal ratings before and after sleep. Significant learning was seen in the SRTT, but this was unrelated to the stimulation condition or the consolidation interval.

One possible explanation for the absence of a reduction in emotional memory recognition after sleep is that theta power during REM sleep actually showed an enhanced relationship with emotional memory after 5 Hz stimulation, while there was no reduction in recognition in this condition. It could be that two opposite effects cancelled each other out: a reduction in emotional memory consolidation during the stimulation period itself, but a subsequent enhancement in the post-stimulation period. This fits with the SWS results, where the absence of a post-stimulation relationship did not interfere with the 0.75 Hz stimulation-related enhancement of neutral memory. If this account is true, it suggests that CtDCS stimulation during sleep may have more complex effects than previously thought, and in particular may have an effect on memory during the stimulation period, and a subsequent effect on neural oscillations during sleep which may themselves have a quite different effect. This is a preliminary finding, however, and requires future research and additional supporting evidence to confirm its significance.

After examining previous research, this is to our knowledge the first study to have applied CtDCS over the PFC during REM sleep to entrain both SO and theta rhythms to subsequently alter memory. Previous research has generally used anodal tDCS, for example, over the PFC during SWS to entrain SO, improving neutral declarative memory performance [65]. The results from the current study show that entrainment is also possible during REM sleep, with the application of tDCS at 0.75 Hz entraining SO and modulating neuronal dynamics [45] by replacing existing oscillatory activity [66] to selectively improve neutral declarative memory [41]. Increasing SO would explain the increase in neutral declarative memory seen in the current study.

Owing to cortical folding in the brain, both anodal and CtDCS result in mixed-field potentials, with a unidirectional neural firing rate increase, suggesting that CtDCS could either inhibit or improve memory [32]. The extent of this increased firing rate may also depend on the differing SWA and theta waveforms, which may explain the differences in performance benefits for the 0.75 Hz and 5 Hz conditions seen in the current study.

SWA is uniquely characterized by up-states involving neural depolarization in which neurons actively fire, as well as down-states involving hyperpolarizing neural silence [5,67]. For CtDCS, there is an alignment of the on-period of stimulation with the down-state (hyperpolarization) of neural firing, the stimulation then attempts to reduce/inhibit hyperpolarizing down-states but as the neurons in the down-state of SWA are already fully hyperpolarized, no further inhibition can occur. As CtDCS cannot inhibit the varied cortical orientation of the CtDCS due to cortical folding, it may improve memory by also aligning with the up-state of neural firing, further depolarizing these [32] and partially explaining the improvement in neutral declarative memory after 0.75 Hz stimulation. This finding has so far been limited to monophasic pulse stimulation as used in the current study, in which hyperpolarizing down-states of the stimulation have been equal to zero [41].

Long-term potentiation is sensitive to theta phase, with theta peaks increasing potentiation and troughs creating large-scale depression [68]. A key difference between the SO and theta, however, is the lack of completely hyperpolarized down-state in theta oscillations [33]. The on-period of the cathodal stimulation, when aligning with the down-state as found within theta troughs may be able to increase this hyperpolarization further, thereby creating an inhibitory effect. However, simultaneous inhibition during theta troughs and improvements during theta peaks may effectively cancel each other out resulting in no memory alteration [32]. This fits with the findings of our current study, where the absence of memory alteration after theta stimulation during REM sleep, in contrast to the clear effect seen for SO stimulation, could be partly due to the simultaneous memory inhibition of theta troughs not found in SO down-states [33]. However, this hypothesis requires further empirical validation.

The alignment of stimulation with up and down-states and subsequent unidirectional neural firing increase could alter homeostatic plasticity resulting in accelerated homeostatic regulation typically shown during sleep [5,8]. According to the synaptic homeostasis hypothesis, global neuronal downscaling occurs during sleep due to a decrease in synaptic strength [69], ensuring that space and energy resources in the brain are not exceeded [70] as during wake synaptic potentiation occurs involving an upscaling of synaptic strength and increased energy costs gained through stimulus learning [8]. Greater downscaling then improves memory consolidation through an enhanced signal-to-noise ratio [71] improving memories more strongly potentiated during wake and eliminating memories weakly potentiated during wake [72]. The process of downscaling can explain why the benefits of SOs for memory consolidation may also occur after artificial stimulation at the SO frequency.

The active systems consolidation hypothesis can potentially explain why neutral declarative memory was preferentially improved during the 0.75 Hz stimulation. SWA during SWS, including SO, aids with system consolidation involving memory reactivations to mediate the transfer of memory representation binding from the hippocampus to the neocortex [6,7]. Owing to a greater reliance on the hippocampus for neutral declarative memories [73], these memories could have been selectively improved through greater reactivation after 0.75 Hz stimulation. Reactivations also protect the synapses from downscaling [74] as reactivation also increases the strength of the memories [5,67]. Performance improvements for neutral declarative memory may therefore have been caused by increased SWA improving system consolidation [6]. The absence of a significant correlation between the duration of SWS is compatible with this explanation, as SWA is driven by homeostatic sleep pressure and not perfectly correlated with SWS [75]. We also use Remember responses for our behavioural measurement as we wanted to focus on the clearest responses which retained contextual information associated with the hippocampus, while SWS may be more strongly related to decontextualization [76]. Together with the absence of a significant correlation with spectral power measures (which excluded the stimulation period), our results strongly suggest that the benefit to neutral declarative memory seen in the 0.75 Hz condition was the result of the increase in SO as a result of the stimulation, and unrelated to existing periods of SWS. This finding is potentially significant, as it confirms that entrainment does not have to take place in the most closely related sleep stage in order to be effective. Based on this, future studies could explore the benefits of increasing SWA using tDCS for insomniacs [77] and explore the potential memory benefits for clinical groups with impaired neutral declarative memory, including Down's syndrome [78].

No significant post-sleep AoL improvement for the SRTT was found after applying either 0.75 or 5 Hz stimulation when compared to the sham condition. This finding is consistent with previous findings examining SRTT performance after applying 5 Hz tDCS to the premotor cortex [56]. The SRTT contains both declarative and procedural elements [46]. The declarative element would suggest a potential benefit of stimulation at 0.75 Hz; however, no such benefit was seen in our study. There are two explanations for this. First, it has been shown that the hippocampal-dependent declarative memory aspects of the SRTT are linked directly with explicit knowledge of the sequence [79,80]. In line with this finding, a previous study found no sleep-dependent consolidation of SRTT sequences unless the task was encoded explicitly, or contextual elements existed within the task [48], and that awareness modifies the sleep-related benefit [81]. Second, it has been shown that inclusion of a declarative memory task immediately after the SRTT disrupts the declarative representation and consolidation of the sequence [82], and in our study the SRTT was followed by a word recognition declarative learning task. Corroboration of this comes from the reaction times in our SRTT task, which stayed fairly consistent between the pre- and post-sleep sessions and did not decrease significantly over time to indicate learning had taken place when previously reaction times of 300 ms were found to decrease to 200 ms when the sequences are explicitly learned [48], it can be reasonably suggested that the sequence remained largely implicit [83]. Our results, therefore, fit with the existing literature in this area and confirm that the SRTT does not benefit from increased SO power in the absence of an explicit component.

The SRTT also contains procedural elements and is often treated as an implicit procedural task [48,56]. This suggest 5 Hz tDCS could also have been beneficial, in the sense that theta-rich REM sleep can improve implicit procedural memories [84], and indeed a positive trend was seen between theta power during REM sleep and the AoL after sleep, but no overall benefit to performance was seen. Procedural memory improvements after REM sleep have been related to synaptic consolidation occurring during this sleep stage, involving an integration of memories with previous knowledge for long-term storage [6]. Consolidation of locally encoded information in the cortico-striatal network [7] has been linked with procedural memory [85], specifically for implicit motor skills [86,87] such as those that may be present in the SRTT. There are two explanations for the absence of an effect seen in our study. First, previous research has suggested that tDCS can alter declarative, but not procedural memories [28]; our findings fit with this suggestion. Various reasons have been proposed for this, including a low current density producing only short-term tDCS effects on memory [56] and stimulation of the PFC instead of the premotor cortex for procedural memories [41]. A systematic investigation of CtDCS stimulation at different scalp locations during sleep is also currently absent from the literature and would make an important future study. The second explanation is the same reason that 5 Hz stimulation did not inhibit negative emotional memory, which is that CtDCS 5 Hz stimulation during REM sleep is not inherently targeting the peaks or troughs of existing theta waves and is likely to be getting a mixture of the two, which largely cancels out the potential benefits [32]. Future studies using advanced real-time feedback technology, as seen in auditory stimulation during sleep [88], could test this hypothesis by presenting electrical stimulation at precise points in the EEG waveform.

## 5. Conclusion

This study is, to our knowledge, the first to demonstrate that CtDCS can enhance memory when applied to specific waveforms at specific frequencies, contrary to the prevailing view that CtDCS exclusively inhibits neural activity [29]. Stimulation at 0.75 Hz during REM sleep, boosting activity in the SO band during the stimulation period, resulted in an improvement for neutral word recognition, probably due to greater SO activity mediating the transfer of memory representations from the hippocampus to the neocortex [6] resulting in improved hippocampal-dependent non-declarative memory specifically [71]. By contrast the hypothesized inhibitory effect of 5 Hz stimulation during REM sleep on negative emotional memory failed to materialize. One reason for this could be that a reduction in consolidation of negative emotional memory during 5 Hz stimulation was offset by a subsequent stronger positive coupling between theta power during REM and emotional memory, leading to two conflicting effects. A second possible reason is that there are different ways in which the stimulation interacts with existing waveforms. Specifically, the inability to further depolarize SO during troughs means that temporally non-targeted stimulation will result in a net increase in excitation. However, theta waves can be depolarized as well as hyperpolarized, so non-targeted stimulation is likely to cancel out and have relatively little net effect [32]. Both of these reasons may also explain the absence of an effect on the procedural elements of the SRTT which we saw. The SRTT was also not enhanced by 0.75 Hz stimulation, largely because the standard implicit version of that task does not seem to benefit from SO during sleep. Future studies should focus on three areas. First, further investigation of the effect of CtDCS during sleep on neural oscillations during stimulation and after stimulation, to consider the possibility that these effects may be different. Second, an exploration of the CtDCS stimulation at different frequencies using advanced technology to control the precise temporal location of the stimulation to test the peaks/troughs hypothesis. Third, studies to test the potential benefits of boosting SO using CtDCS in clinical populations in need of greater SWA or neutral declarative memory.

## Data accessibility

All of the data for this experiment, in a usable data sheet format suitable for replicating all of the analyses included here and potentially conducting others, has been made available and can be downloaded from: http://sleep.blogs.lincoln.ac.uk/tdcs-data-page. The data have been made available in both its original format and an open format to facilitate data sharing. It can be analysed using SPSS (commercial, available from: https://www.ibm.com/analytics/data-science/predictive-analytics/spss-statistical-software) or R (free software, available from https://www.r-project.org/).

## References

[1] Born J, Wilhelm I 2012 System consolidation of memory during sleep. Psychol. Res. 76, 192–203. (10.1007/s00426-011-0335-6) 21541757 PMC3278619

[2] Walker MP, Stickgold R 2004 Sleep-dependent learning and memory consolidation. Neuron 44, 121–133. (10.1016/j.neuron.2004.08.031) 15450165

[3] Marshall L, Born J 2007 The contribution of sleep to hippocampus-dependent memory consolidation. Trends Cogn. Sci. 11, 442–450. (10.1016/j.tics.2007.09.001) 17905642

[4] Stickgold R, Walker MP 2007 Sleep-dependent memory consolidation and reconsolidation. Sleep Med. 8, 331–343. (10.1016/j.sleep.2007.03.011) 17470412 PMC2680680

[5] Tononi G, Cirelli C 2014 Sleep and the price of plasticity: from synaptic and cellular homeostasis to memory consolidation and integration. Neuron 81, 12–34. (10.1016/j.neuron.2013.12.025) 24411729 PMC3921176

[6] Rasch B, Born J 2013 About sleep's role in memory. Physiol. Rev. 93, 681–766. (10.1152/physrev.00032.2012) 23589831 PMC3768102

[7] Diekelmann S, Born J 2010 The memory function of sleep. Nat. Rev. Neurosci. 11, 114–126. (10.1038/nrn2762) 20046194

[8] Tononi G, Cirelli C 2006 Sleep function and synaptic homeostasis. Sleep Med. Rev. 10, 49–62. (10.1016/j.smrv.2005.05.002) 16376591

[9] Steriade M, Timofeev I 2003 Neuronal plasticity in thalamocortical networks during sleep and waking oscillations. Neuron 37, 563–576. (10.1016/S0896-6273(03)00065-5) 12597855

[10] Alger SE, Lau H, Fishbein W 2012 Slow wave sleep during a daytime nap is necessary for protection from subsequent interference and long-term retention. Neurobiol. Learn. Mem. 98, 188–196. (10.1016/j.nlm.2012.06.003) 22732649

[11] Wilhelm I, Diekelmann S, Molzow I, Ayoub A, Mölle M, Born J 2011 Sleep selectively enhances memory expected to be of future relevance. J. Neurosci. 31, 1563–1569. (10.1523/JNEUROSCI.3575-10.2011) 21289163 PMC6623736

[12] Nissen MJ, Bullemer P 1987 Attentional requirements of learning: evidence from performance measures. Cognit. Psychol. 19, 1–32. (10.1016/0010-0285(87)90002-8)

[13] Spencer RM, Sunm M, Ivry RB 2006 Sleep-dependent consolidation of contextual learning. Curr. Biol. 16, 1001–1005. (10.1016/j.cub.2006.03.094) 16713957

[14] Groch S, Wilhelm I, Diekelmann S, Born J 2013 The role of REM sleep in the processing of emotional memories: evidence from behavior and event-related potentials. Neurobiol. Learn. Mem. 99, 1–9. (10.1016/j.nlm.2012.10.006) 23123802

[15] Genzel L, Spoormaker VI, Konrad BN, Dresler M 2015 The role of rapid eye movement sleep for amygdala-related memory processing. Neurobiol. Learn. Mem. 122, 110–121. (10.1016/j.nlm.2015.01.008) 25638277

[16] Wiesner CD, Pulst J, Krause F, Elsner M, Baving L, Pedersen A, Prehn-Kristensen A, Göder R 2015 The effect of selective REM-sleep deprivation on the consolidation and affective evaluation of emotional memories. Neurobiol. Learn. Mem. 122, 131–141. (10.1016/j.nlm.2015.02.008) 25708092

[17] Hutchison IC, Rathore S 2015 The role of REM sleep theta activity in emotional memory. Front. Psychol. 6, 1439–1454. (10.3389/fpsyg.2015.01439) 26483709 PMC4589642

[18] Kensinger EA, Schacter DL 2008 Memory and emotion. In Handbook of emotions (eds Lewis M, Haviland-Jones JM, Barrett LF), pp. 601–617. New York, NY: The Guilford Press.

[19] Murty VP, Ritchey M, Adcock RA, LaBar KS 2010 fMRI studies of successful emotional memory encoding: a quantitative meta-analysis. Neuropsychologia 48, 3459–3469. (10.1016/j.neuropsychologia.2010.07.030) 20688087 PMC2949536

[20] McGaugh JL 2004 The amygdala modulates the consolidation of memories of emotionally arousing experiences. Annu. Rev. Neurosci. 27, 1–28. (10.1146/annurev.neuro.27.070203.144157) 15217324

[21] Davidson RJ, Tomarken AJ 1989 Laterality and emotion: an electrophysiological approach. In Handbook of neuropsychology (eds Boiler F, Grafman J), pp. 419–441. Amsterdam, The Netherlands: Elsevier.

[22] Grimm S, Schmidt CF, Bermpohl F, Heinzel A, Dahlem Y, Wyss M, Hell D, Boesiger P, Northoff G 2006 Segregated neural representation of distinct emotion dimensions in the prefrontal cortex—an fMRI study. Neuroimage 30, 325–340. (10.1016/j.neuroimage.2005.09.006) 16230029

[23] Penolazzi B, Di Domenico A, Marzoli D, Mammarella N, Fairfield B, Franciotti R, Brancucci A, Tommasi L 2010 Effects of transcranial direct current stimulation on episodic memory related to emotional visual stimuli. PLoS ONE 5, e10623 (10.1371/journal.pone.0010623) 20498700 PMC2869343

[24] Adolphs R, Jansari A, Tranel D 2001 Hemispheric perception of emotional valence from facial expressions. Neuropsychology 15, 516–524. (10.1037/0894-4105.15.4.516) 11761041

[25] Nitsche MA, Koschack J, Pohlers H, Hullemann S, Paulus W, Happe S 2012 Effects of frontal transcranial direct current stimulation on emotional state and processing in healthy humans. Front. Psychiatry 3, 58–68. (10.3389/fpsyt.2012.00058) 22723786 PMC3377009

[26] Hecht D 2010 Depression and the hyperactive right-hemisphere. Neurosci. Res. 2, 77–87. (10.1016/j.neures.2010.06.013) 20603163

[27] Boggio PS, Bermpohl F, Vergara AO, Muniz AL, Nahas FH, Leme PB, Rigonatti SP, Fregni F 2007 Go-no-go task performance improvement after anodal transcranial DC stimulation of the left dorsolateral prefrontal cortex in major depression. J. Affect. Disord. 101, 91–98. (10.1016/j.jad.2006.10.026) 17166593

[28] Barham MP, Enticott PG, Conduit R, Lum JA 2016 Transcranial electrical stimulation during sleep enhances declarative (but not procedural) memory consolidation: evidence from a meta-analysis. Neurosci. Biobehav. Rev. 63, 65–77. (10.1016/j.neubiorev.2016.01.009) 26828569

[29] Nitsche MA et al. 2008 Transcranial direct current stimulation: state of the art 2008. Brain Stimulat. 1, 206–223. (10.1016/j.brs.2008.06.004) 20633386

[30] Nitsche MA, Liebetanz D, Antal A, Lang N, Tergau F, Paulus W 2003 Modulation of cortical excitability by weak direct current stimulation—technical, safety and functional aspects. Suppl. Clin. Neurophysiol. 56, 255–276. (10.1016/S1567-424X(09)70230-2) 14677403

[31] Herrmann CS, Rach S, Neuling T, Strüber D 2013 Transcranial alternating current stimulation: a review of the underlying mechanisms and modulation of cognitive processes. Front. Hum. Neurosci. 7, 279 (10.3389/fnhum.2013.00279) 23785325 PMC3682121

[32] Reato D, Rahman A, Bikson M, Parra LC 2013 Effects of weak transcranial alternating current stimulation on brain activity—a review of known mechanisms from animal studies. Front. Hum. Neurosci. 7, 687–695. (10.3389/fnhum.2013.00687) 24167483 PMC3805939

[33] Headley DB, Paré D 2017 Common oscillatory mechanisms across multiple memory systems. npj Science of Learning 2, 1 (10.1038/s41539-016-0001-2) PMC6171763 30294452

[34] Balzarotti S, Colombo B 2016 Effects of unilateral transcranial direct current stimulation of left prefrontal cortex on processing and memory of emotional visual stimuli. PLoS ONE 11, e0159555 (10.1371/journal.pone.0159555) 27433807 PMC4951131

[35] Nishida M, Pearsall J, Buckner RL, Walker MP 2008 REM sleep, prefrontal theta, and the consolidation of human emotional memory. Cereb. Cortex 19, 1158–1166. (10.1093/cercor/bhn155) 18832332 PMC2665156

[36] Wagner U, Gais S, Born J 2001 Emotional memory formation is enhanced across sleep intervals with high amounts of rapid eye movement sleep. Learn. Mem. 8, 112–119. (10.1101/lm.36801) 11274257 PMC311359

[37] Weigand A, Richtermeier A, Feeser M, Guo JS, Briesemeister BB, Grimm S, Bajbouj M 2013 State-dependent effects of prefrontal repetitive transcranial magnetic stimulation on emotional working memory. Brain Stimulat. 6, 905–912. (10.1016/j.brs.2013.06.004) 23928102

[38] Morgan HM, Davis NJ, Bracewell RM 2014 Does transcranial direct current stimulation to prefrontal cortex affect mood and emotional memory retrieval in healthy individuals? PLoS ONE 9, e92162 (10.1371/journal.pone.0092162) 24651375 PMC3961298

[39] Sahlem GL et al. 2015 Oscillating square wave transcranial direct current stimulation (tDCS) delivered during slow wave sleep does not improve declarative memory more than sham: a randomized sham controlled crossover study. Brain Stimulat. 8, 528–534. (10.1016/j.brs.2015.01.414) PMC4598642 25795621

[40] Eggert T, Dorn H, Sauter C, Nitsche MA, Bajbouj M, Danker-Hopfe H 2013 No effects of slow oscillatory transcranial direct current stimulation (tDCS) on sleep-dependent memory consolidation in healthy elderly subjects. Brain Stimulat. 6, 938–945. (10.1016/j.brs.2013.05.006) 23810208

[41] Marshall L, Kirov R, Brade J, Mölle M, Born J 2011 Transcranial electrical currents to probe EEG brain rhythms and memory consolidation during sleep in humans. PLoS ONE 6, e16905 (10.1371/journal.pone.0016905) 21340034 PMC3038929

[42] Westerberg CE, Florczak SM, Weintraub S, Mesulam MM, Marshall L, Zee PC, Paller KA 2015 Memory improvement via slow-oscillatory stimulation during sleep in older adults. Neurobiol. Aging 36, 2577–2586. (10.1016/j.neurobiolaging.2015.05.014) 26116933 PMC4523433

[43] Göder R, Baier PC, Beith B, Baecker C, Seeck-Hirschner M, Junghanns K, Marshall L 2013 Effects of transcranial direct current stimulation during sleep on memory performance in patients with schizophrenia. Schizophr. Res. 144, 153–154. (10.1016/j.schres.2012.12.014) 23336963

[44] Marshall L, Helgadottir H, Molle M, Born J 2006 Boosting slow oscillations during sleep potentiates memory. Nature 444, 610–613. (10.1038/nature05278) 17086200

[45] Fröhlich F, McCormick DA 2010 Endogenous electric fields may guide neocortical network activity. Neuron 67, 129–143. (10.1016/j.neuron.2010.06.005) 20624597 PMC3139922

[46] Robertson EM 2007 The serial reaction time task: implicit motor skill learning? J. Neurosci. 27, 10 073–10 075. (10.1523/JNEUROSCI.2747-07.2007) PMC6672677 17881512

[47] Maquet P et al. 2000 Experience-dependent changes in cerebral activation during human REM sleep. Nat. Neurosci. 3, 831–836. (10.1038/77744) 10903578

[48] Spencer RM, Gouw AM, Ivry RB 2007 Age-related decline of sleep-dependent consolidation. Learn. Mem. 14, 480–484. (10.1101/lm.569407) 17622650

[49] Kleiner M, Brainard D, Pelli D, Ingling A, Murray R, Broussard C 2007 What's new in Psychtoolbox-3? Perception 36, 1–16. (10.1068/v070821)

[50] Van Overschelde JP, Rawson KA, Dunlosky J 2004 Category norms: an updated and expanded version of the norms. J. Mem. Lang. 50, 289–335. (10.1016/j.jml.2003.10.003)

[51] Yonelinas A 2002 The nature of recollection and familiarity: a review of 30 years of research. J. Mem. Lang. 46, 441–517. (10.1006/jmla.2002.2864)

[52] Reed J, Johnson P 1994 Assessing implicit learning with indirect tests: determining what is learned about sequence structure. J. Exp. Psychol. Learn. Mem. Cogn. 20, 585–594. (10.1037/0278-7393.20.3.585)

[53] Destrebecqz A, Cleeremans A 2001 Can sequence learning be implicit? New evidence with the process dissociation procedure. Psychon. Bull. Rev. 8, 343–350. (10.3758/BF03196171) 11495124

[54] Bikson M et al. 2016 Safety of transcranial direct current stimulation: evidence based update 2016. Brain Stimulat. 9, 641–661. (10.1016/j.brs.2016.06.004) PMC5007190 27372845

[55] Boukadoum AM, Ktonas PY 1988 Non-random patterns of REM occurrences during REM sleep in normal human subjects: an automated second-order study using Markovian modeling. Electroencephalogr. Clin. Neurophysiol. 70, 404–416. (10.1016/0013-4694(88)90018-1) 2460314

[56] Nitsche MA, Jakoubkova M, Thirugnanasambandam N, Schmalfuss L, Hullemann S, Sonka K, Trenkwalder C, Happe S 2010 Contribution of the premotor cortex to consolidation of motor sequence learning in humans during sleep. J. Neurophysiol. 104, 2603–2614. (10.1152/jn.00611.2010) 20844115

[57] Reinhart RMG, Cosman JD, Fukuda K, Woodman GF 2017 Using transcranial direct-current stimulation (tDCS) to understand cognitive processing. Attent. Percept. Psychophys. 79, 3–23. (10.3758/s13414-016-1224-2) PMC5539401 27804033

[58] Homan RW, Herman J, Purdy P 1987 Cerebral location of international 10–20 system electrode placement. Electroencephalogr. Clin. Neurophysiol. 66, 376–382. (10.1016/0013-4694(87)90206-9) 2435517

[59] Iber C, American Academy of Sleep Medicine. 2007 The AASM manual for the scoring of sleep and associated events: rules, terminology and technical specifications. Westchester, IL: American Academy of Sleep Medicine.

[60] Hoddes E, Dement W, Zarcone V 1972 The development and use of the Stanford sleepiness scale (SSS). Psychophysiology 9, 150 (10.1111/j.1469-8986.1972.tb00747.x)

[61] Neath K 2012 The use of facial features in facial expression discrimination. Unpublished doctoral dissertation, University of Waterloo, Canada.

[62] Delorme A, Makeig S 2004 EEGLAB: an open source toolbox for analysis of single-trial EEG dynamics including independent component analysis. J. Neurosci. Methods 134, 9–21. (10.1016/j.jneumeth.2003.10.009) 15102499

[63] Welch P 1967 The use of fast Fourier transform for the estimation of power spectra: a method based on time averaging over short, modified periodograms. IEEE Trans. Audio Electroacoust. 15, 17–20. (10.1109/TAU.1967.1161901)

[64] Born J 2010 Slow-wave sleep and the consolidation of long-term memory. World J. Biol. Psychiatry 11, 16–21. (10.3109/15622971003637637) 20509828

[65] Marshall L, Mölle M, Hallschmid M, Born J 2004 Transcranial direct current stimulation during sleep improves declarative memory. J. Neurosci. 24, 9985–9992. (10.1523/JNEUROSCI.2725-04.2004) 15525784 PMC6730231

[66] Ali MM, Sellers KK, Fröhlich F 2013 Transcranial alternating current stimulation modulates large-scale cortical network activity by network resonance. J. Neurosci. 33, 11 262–11 275. (10.1523/JNEUROSCI.5867-12.2013) PMC6618612 23825429

[67] Chauvette S, Crochet S, Volgushev M, Timofeev I 2011 Properties of slow oscillation during slow-wave sleep and anesthesia in cats. J. Neurosci. 31, 14 998–15 008. (10.1523/JNEUROSCI.2339-11.2011) PMC3209581 22016533

[68] Bikbaev A, Manahan-Vaughan D 2008 Relationship of hippocampal theta and gamma oscillations to potentiation of synaptic transmission. Front. Neurosci. 2, 56–63. (10.3389/neuro.01.010.2008) 18982107 PMC2570077

[69] Liu ZW, Faraguna U, Cirelli C, Tononi G, Gao XB 2010 Direct evidence for wake-related increases and sleep-related decreases in synaptic strength in rodent cortex. J. Neurosci. 30, 8671–8675. (10.1523/JNEUROSCI.1409-10.2010) 20573912 PMC2903226

[70] Born J, Feld GB 2012 Sleep to upscale, sleep to downscale: balancing homeostasis and plasticity. Neuron 75, 933–935. (10.1016/j.neuron.2012.09.007) 22998858

[71] Nere A, Hashmi A, Cirelli C, Tononi G 2013 Sleep-dependent synaptic down-selection (I): modeling the benefits of sleep on memory consolidation and integration. Front. Neurol. 4, 143 (10.3389/fneur.2013.00143) 24137153 PMC3786405

[72] Ellenbogen JM, Hu PT, Payne JD, Titone D, Walker MP 2007 Human relational memory requires time and sleep. Proc. Natl Acad. Sci. USA 104, 7723–7728. (10.1073/pnas.0700094104) 17449637 PMC1863467

[73] Mölle M, Born J 2009 Hippocampus whispering in deep sleep to prefrontal cortex—for good memories? Neuron 61, 496–498. (10.1016/j.neuron.2009.02.002.) 19249269

[74] Wang G, Grone B, Colas D, Appelbaum L, Mourrain P 2011 Synaptic plasticity in sleep: learning, homeostasis and disease. Trends Neurosci. 34, 452–463. (10.1016/j.tins.2011.07.005) 21840068 PMC3385863

[75] Dijk DJ, Czeisler CA 1995 Contribution of the circadian pacemaker and the sleep homeostat to sleep propensity, sleep structure, electroencephalographic slow waves, and sleep spindle activity in humans. J. Neurosci. 15, 3526–3538. (10.1523/JNEUROSCI.15-05-03526.1995) 7751928 PMC6578184

[76] Inostroza M, Born J 2013 Sleep for preserving and transforming episodic memory. Annu. Rev. Neurosci. 36, 79–102. (10.1146/annurev-neuro-062012-170429) 23642099

[77] Walsh JK 2009 Enhancement of slow wave sleep: implications for insomnia. J. Clin. Sleep Med. 5, 27–32. PMC2824211 19998872

[78] Jarrold C, Nadel L, Vicari S 2009 Memory and neuropsychology in down syndrome. Down Syndr. Res. Pract. 12, n3 (10.3104/reviews/2068)

[79] Du Y, Prashad S, Schoenbrun I, Clark JE 2016 Probabilistic motor sequence yields greater offline and less online learning than fixed sequence. Front. Hum. Neurosci. 10, 87 (10.3389/fnhum.2016.00087) 26973502 PMC4773591

[80] Destrebecqz A 2004 The effect of explicit knowledge on sequence learning: a graded account. Psychol. Belg. 44, 217–247. (10.5334/pb-44-4-217)

[81] Robertson EM, Pascual-Leone A, Press DZ 2004 Awareness modifies the skill-learning benefits of sleep. Curr. Biol. 14, 208–212. (10.1016/s0960-9822(04)00039-9) 14761652

[82] Brown RM, Robertson EM 2007 Off-line processing: reciprocal interactions between declarative and procedural memories. J. Neurosci. 27, 10 468–10 475. (10.1523/jneurosci.2799-07.2007) PMC6673170 17898218

[83] Morehead JR, Butcher PA, Taylor JA 2011 Does fast learning depend on declarative mechanisms? J. Neurosci. 31, 5184–5185. (10.1523/JNEUROSCI.0040-11.2011) 21471352 PMC6622709

[84] Yordanova J, Kolev V, Verleger R, Bataghva Z, Born J, Wagner U 2008 Shifting from implicit to explicit knowledge: different roles of early- and late-night sleep. Learn. Mem. 15, 508–515. (10.1101/lm.897908) 18626095 PMC2505318

[85] Doyon J, Penhune V, Ungerleider LG 2003 Distinct contribution of the cortico-striatal and cortico-cerebellar systems to motor skill learning. Neuropsychologia 41, 252–262. (10.1016/S0028-3932(02)00158-6) 12457751

[86] Clark GM, Lum JA, Ullman MT 2014 A meta-analysis and meta-regression of serial reaction time task performance in Parkinson's disease. Neuropsychology 28, 945–958. (10.1037/neu0000121) 25000326

[87] Hardwick RM, Rottschy C, Miall RC, Eickhoff SB 2013 A quantitative meta-analysis and review of motor learning in the human brain. Neuroimage 67, 283–297. (10.1016/j.neuroimage.2012.11.020) 23194819 PMC3555187

[88] Ngo HVV, Martinetz T, Born J, Mölle M 2013 Auditory closed-loop stimulation of the sleep slow oscillation enhances memory. Neuron 78, 545–553. (10.1016/j.neuron.2013.03.006) 23583623

